# Supplementary material for: What do doctors understand by spiritual health? A survey of UK general practitioners
Source: BMJ Open. 2021 Aug 11;11(8):e045110. doi: 10.1136/bmjopen-2020-045110 (PMC8359514; doi:10.1136/bmjopen-2020-045110)
Supplement: Author's manuscript [file bmjopen-2020-045110.draft_revisions.pdf]

BMJ Open

BMJ Open is committed to open peer review. As part of this commitment we make the peer review history of every article we publish publicly available.

When an article is published we post the peer reviewers' comments and the authors' responses online. We also post the versions of the paper that were used during peer review. These are the versions that the peer review comments apply to.

The versions of the paper that follow are the versions that were submitted during the peer review process. They are not the versions of record or the final published versions. They should not be cited or distributed as the published version of this manuscript.

BMJ Open is an open access journal and the full, final, typeset and author-corrected version of record of the manuscript is available on our site with no access controls, subscription charges or pay-per-view fees (http://bmjopen.bmj.com).

If you have any questions on BMJ Open's open peer review process please email info.bmjopen@bmj.com

BMJ Open

What Do Doctors Understand By 'Spiritual Health'? A survey of UK general practitioners

Journal:	*BMJ Open*
Manuscript ID	bmjopen-2020-045110
Article Type:	Original research
Date Submitted by the Author:	28-Sep-2020

Complete List of Authors:	Whitehead, Orla; Newcastle University, Population Health Sciences Institute<br>Jagger, Carol; University of Newcastle<br>Hanratty, Barbara; Newcastle University, Institute of Health and Society / Newcastle University Institute for Ageing

Keywords:	PRIMARY CARE, SOCIAL MEDICINE, PSYCHIATRY, PALLIATIVE CARE

SCHOLARONE™ Manuscripts

I, the Submitting Author has the right to grant and does grant on behalf of all authors of the Work (as defined in the below author licence), an exclusive licence and/or a non-exclusive licence for contributions from authors who are: i) UK Crown employees; ii) where BMJ has agreed a CC-BY licence shall apply, and/or iii) in accordance with the terms applicable for US Federal Government officers or employees acting as part of their official duties; on a worldwide, perpetual, irrevocable, royalty-free basis to BMJ Publishing Group Ltd ("BMJ") its licensees and where the relevant Journal is co-owned by BMJ to the co-owners of the Journal, to publish the Work in this journal and any other BMJ products and to exploit all rights, as set out in our licence.

The Submitting Author accepts and understands that any supply made under these terms is made by BMJ to the Submitting Author unless you are acting as an employee on behalf of your employer or a postgraduate student of an affiliated institution which is paying any applicable article publishing charge ("APC") for Open Access articles. Where the Submitting Author wishes to make the Work available on an Open Access basis (and intends to pay the relevant APC), the terms of reuse of such Open Access shall be governed by a Creative Commons licence – details of these licences and which Creative Commons licence will apply to this Work are set out in our licence referred to above.

Other than as permitted in any relevant BMJ Author's Self Archiving Policies, I confirm this Work has not been accepted for publication elsewhere, is not being considered for publication elsewhere and does not duplicate material already published. I confirm all authors consent to publication of this Work and authorise the granting of this licence.

For peer review only

TITLE: What do doctors understand by spiritual health? A survey of UK general practitioners

Authors : Orla Whitehead, Carol Jagger, Barbara Hanratty

orla.whitehead@newcastle.ac.uk, carol.jagger@newcastle.ac.uk, barbara.hanratty@newcastle.ac.uk

Keywords: Spirituality, General Practitioners, Consensus, United Kingdom, Religion, Spiritual Therapies, Surveys and Questionnaires, Referral and Consultation, Spiritual Health.

Word count: 2468

Corresponding author: Dr Orla Whitehead, Newcastle University, Biomedical Research Building, Campus for Ageing and Vitality, Newcastle upon Tyne, NE4 5PL orla.whitehead@newcastle.ac.uk

For peer review only

ABSTRACT

Objective:

To explore how GPs understand and define the term ‘spiritual health’.

Design:

An online survey asked practicing GPs in England about discussing spiritual health with patients. Qualitative free text responses on how GPs defined spiritual health and additional free text comments were subject to thematic analysis.

Setting:

Primary Care in England, 2019.

Participants:

177 practicing GPs, 95% trained in England, 63% female, 79% white British, 63% Christian.

Results

177 GPs responded to the survey, and most (69%) respondents considered themselves to be spiritual people. Just under half (48%) considered themselves religious, and one fifth of respondents (21%) reported that they were spiritual, not religious. Understanding of spiritual health fitted into three themes: self-actualisation and meaning, transcendence and relationships beyond the self, and expressions of spirituality. A full range of views were expressed, from a minority who challenged their role in spiritual health,through to others enthusiastic about its place in health care.

For peer review only - http://bmjopen.bmj.com/site/about/guidelines.xhtml

Conclusion

Spiritual health and religiosity are understood by English GPs to be distinct concepts. A consensus definition of spiritual health incorporating the themes identified by working doctors, may be helpful to support GPs to follow the recommended guidance in their practice.

ARTICLE SUMMARY

Strengths and Limitations

- Spiritual health is a nebulous, undefined concept, but UK doctors have it within their professional remit. This survey offers a threefold definition of spiritual health developed by family doctors.
- This is the largest survey of doctors in Europe on the topic of spiritual health, as 177 practicing GPs responded, a large number from a group who are often difficult to engage in research. A broad range of views was expressed, suggesting that selection bias was minimal.
- Data were collected on religious views, despite this being a potentially sensitive topic.

For peer review only - http://bmjopen.bmj.com/site/about/guidelines.xhtml

INTRODUCTION

Modern medicine may have little time for the spiritual aspects of life, however, healers have always been required to address more than physical problems.[1] In present day secular societies, interest in holistic care and mental wellbeing is growing.[2] Holism underpins primary care, and general practitioners (GPs) are expected to treat patients within their social and cultural context.[3] The UK regulatory body, the General Medical Council (GMC), expects doctors to consider the religious, social and cultural aspects of a patient's problem,[4] and the Royal College of General Practitioners (RCGP) includes spiritual health in their curriculum for GP trainees.[3] National Institute for Health and Care Excellence guidance also recommends that discussion of spiritual beliefs should be part of the care of dying patients.[5] Discussing spiritual health may be a beneficial intervention for patients,[6] and there has been speculation that it could increase job satisfaction for GPs.[7] Spirituality can also be a resource for health and wellbeing. Religiosity and spirituality are associated with longevity,[8 9] and unmet spiritual needs may be detrimental to health.[10] Research into interactions between spirituality and health is a growing field.[8 11] In recent years, arguments have been made for the importance of embedding spiritual health within general practice.[12] However, the interface between spirituality and medicine can be controversial.[13] Pathological spiritual and religious beliefs may develop, and some UK GPs who shared their faith with patients have been investigated by the GMC.[14]

There is no consensus or widely accepted definition of spiritual health, despite its proposed place in GPs' work. Spirituality means different things to different people, and understanding may shift across the lifecourse.[15] Some definitions invoke the symptomatology of mental illness, which causes confusion and negative attitudes to the concept.[16 17] Danish authors have argued that the term 'existential health' is more appropriate for a secular multicultural society,[18] while others see existential and spiritual health as synonymous.[19] Similarly, spirituality and religiosity are often conflated.[20-24] For some, spirituality is personality trait predisposing to concern with the metaphysical[25] that has played an important role in human societies and medicine.[26] Previous quantitative survey work with GP trainers in Scotland found that spirituality was perceived by educators as a meaningful, useful, but unclear concept.[27] Researchers investigating how spiritual health should be discussed in primary care have adopted a variety of definitions drawing themes from traditional religion through to humanism (Box 1). One of the most frequently adopted is a consensus definition

For peer review only - http://bmjopen.bmj.com/site/about/guidelines.xhtml

from international holistic care conferences that encompasses all the concepts listed in Box 1, apart from inner peace, strength and resilience. [27 28 29]

A clear understanding of spiritual health will be essential, if it is to become a routine part of holistic care in general practice. This study aims to address this gap, with a qualitative exploration of how GPs understand and define spiritual health in their day to day practice.

Insert Box 1 here (Definitions of spiritual health used in research)

For peer review only

METHOD

Design

An online survey was developed and used to investigate GP views on discussing spiritual health with their patients. (Data on the use of structured tools to support discussions within consultations are reported elsewhere). Information collected from the participants included demographic characteristics (sex, ethnicity and religion); geographical region and years of work experience. Participants were asked to rate themselves on a five point Likert scale, in response to three statements: I am a spiritual person; I am a religious person and I am spiritually healthy. Spiritual health was deliberately undefined in the survey, as GPs were asked 'what does the term spiritual health mean to you?' and invited to share any other thoughts on this topic.

The survey was sent to qualified GPs in England who were working in standard general practice settings, out of hours, and urgent care. People who were temporarily out of practice (for example maternity leave) were encouraged to participate, but those who had retired and would not return to practice were excluded. The survey was sent via Clinical Commissioning Group (CCG) lists, CCG newsletters, professional groups, and practice managers in Spring/Summer 2019, all 211 CCGs were approached.

Data Analysis

Free text responses to the question 'what does the term spiritual health mean to you', were analysed using a deductive thematic analysis, based upon a priori themes from the literature (Box 1). The analysis process followed a standard path: reading and rereading of the data, coding based upon key words and ideas expressed by respondents, comparing and contrasting of similar codes, aggregation into topics that were then grouped into the a priori

For peer review only - http://bmjopen.bmj.com/site/about/guidelines.xhtml

themes, and any other additional themes.[30] These themes were reviewed to develop the central concepts within the data. Deviant cases were examined, to identify insights from respondents most and least comfortable with the topic. Qualitative software was not used to manage these data, rather the concepts were manipulated using coloured pens, paper and scissors. The researchers were cognisant of their own identities, cultures and religious backgrounds in their approach to the topic. Feedback was sought from GPs from other backgrounds in the survey development. Authors 1 and 3 are practicing GPs.

A similar process was followed to analyse the free text provided in the space for additional comments. In this case, GP responses were also mapped against the attitudes to spiritual care identified in Appleby and colleagues' quantitative work with Scottish GP trainers. They identified four categories of attitudes labelled as rejecting, guarded, pragmatic and embracing.[27]

Patient and Public Involvement

Feedback on the initial survey, and subsequent analysis, was sought via the VOICE North group. The group had diverse opinions on the topic, but all recognised the need for holistic care, and the utility of a working definition of 'spiritual health'.

RESULTS

177 practicing GPs responded to the survey, shown in table 1. The majority were female, of white British origin, and with a primary medical qualification obtained in the UK. Most respondents (69%) considered themselves to be spiritual people. Just under half (48%) considered themselves religious, 70% of respondents reported that they had a religion (63% Christian, 7% named other religions). One fifth of respondents (21%) reported that they were spiritual, not religious. Of the 85 respondents who agreed they were religious, none disagreed that they were spiritual people.

Insert Table 1 here (Characteristics of respondents and self reported spirituality and religiosity

For peer review only - http://bmjopen.bmj.com/site/about/guidelines.xhtml

157 out of 177 participants provided a free text response to the question 'what does the term spiritual health' mean to you?' These responses, ranging in length from 1-105 words, were subject to a thematic analysis. Most of the definitions and understanding of spiritual health could be coded into the three a priori themes: 1) self-actualisation and meaning, 2) transcendence and relationships beyond the self, and 3) expressions of spirituality. One additional theme was developed, and labelled 4) 'meaningless'. Religious participation cut across all three of these themes; development of the self is included in many world religions, as is transcendence, community and relationships to other humans, the world, and/or the divine, and of course expression of beliefs. Only one respondent used the term existential health, and a small number of respondents commented that spiritual health is synonymous with mental health. In the description of themes below, direct quotations from participants are included within the text without attribution.

Self-Actualisation and Meaning

Self-actualisation and meaning brought together ideas around humanity/human experience, a sense of spirit or soul, inner strength, peace and resilience and meaning, purpose or self-actualisation. Acceptance of the self, the situation, with a sense of peace and meaning to life was mentioned, both with and without reference to God. Participants referred to the health of a soul, or inner self, which could be either peaceful or disrupted with concerns, conflict, guilt, or "dread about punishment in an afterlife". A sense of purpose was viewed as giving fulfilment and meaning to life, or as an understanding of the identity: "Understanding where I have come from and where I am heading." Personal resilience and inner strength were mentioned as a healthy outcome of acceptance of the soul, of our humanity, and of our status as 'finite beings.' One participant mentioned "security in who we are, and why we're here", expressing that sense of personal identity and purpose that appears to be part of the human experience. Looking into the self, and something of the individual that is "neither physical nor mental", and finding peace and meaning there, is core to this concept. However, few participants mentioned ideas without going on to refer to the position of the individual and their self-actualisation within the world/universe, "understanding your own place and role within society."

For peer review only - http://bmjopen.bmj.com/site/about/guidelines.xhtml

Transcendence and Relationship beyond the Self

This theme encompassed ideas of connections to community, nature and family, with relationship with the divine and sacred. A majority of participants mentioned relationship and connections to the community, 'something bigger', family, and the world/nature, "Being in tune with people. Enjoying and respecting the natural world." Participants referred to: "believing in something bigger than just you," and "connectedness to the world they can't see." Participants were often keen to be inclusive in their definitions, referring to "being comfortable with the world around you and any faith entity you believe in", or referring to "a higher being" rather than a specific deity. Some participants were clear that, for them, this was part of an accepted religious framework, for example "a relationship with Jesus" or "myself as a Child of God". Looking outwards, "the state of one's capacity for love" and a belonging to something outside the self, appears to underpin this concept.

Expression of Spirituality

The concepts of spirituality of the self, and of our connections outside the self, come together into expressions of spirituality, such as values, rituals, practices, and lifestyles. One participant stated "it embraces everything from mindfulness to religious practice (short of pathological religious delusion.)" Many participants mentioned having 'faith', although what this was faith in was not always expanded upon. The freedom to be "able and …visibly practice my faith" was mentioned by one participants, suggesting that spiritual health requires freedom of expression to be permitted. The use of morals, and personal ethics to influence behaviour, for example "believing in something abstract that guides behaviour and thinking", was mentioned as part of spiritual health. The expression of these values and morals was often linked to membership and belonging to a community, or the seeking of self-actualisation.

Meaningless

A small minority of respondents (6%) reported that spiritual health conveyed no meaning, or was unclear, to them, but there was little elaboration on this assertion.

Attitudes to spiritual care

Respondents were invited to add any other thoughts on spiritual health and care in general. A majority contributed their views, and data relating to understanding and definition of spiritual health were mapped to an existing framework developed by Appleby and colleagues in

For peer review only - http://bmjopen.bmj.com/site/about/guidelines.xhtml

Table 2. This is derived from a quantitative survey and presents a categorisation of the spectrum of attitudes to spiritual care. The perceived need for cues by a large number of respondents, and the low numbers of respondents who fit into the 'rejecting' attitude, would suggest that most respondents fall in to the "guarded" and/or "pragmatic" attitudes identified.

Table 2 here

Attitudes to spiritual care: GP views mapped onto a framework of enthusiasm for spiritual care

For peer review only

For peer review only - http://bmjopen.bmj.com/site/about/guidelines.xhtml

DISCUSSION

This large study of practicing UK GPs suggests that spiritual health has a meaning for many doctors, but that this meaning is distinct from religion. Three themes were identified to encompass GPs' understanding of spiritual health: self-actualisation and meaning, transcendence and relationships beyond the self, and expression of spirituality. Difficulty in articulating exactly what is conveyed by the term spiritual health was a common challenge.

Recruiting GPs via CCG newsletters and other forms of publicity resulted in an good sample size, but provides no information on people who decided not to take part. The range of views that were expressed provides some confidence in our approach, but we acknowledge that people with strong views from either the "rejecting" or "embracing" ends of the spectrum may be more likely to have participated. Respondents were drawn from across England, with a majority in the north. This was probably due to greater engagement with the project from CCGs in the area local to the research team.

Respondents differed from the UK GP population registered with the GMC, which should be considered alongside the findings. Compared to the GP population registered with the GMC, respondents were more likely to be female (63% vs 53%) and to have a medical qualification from the UK (89% vs 79%), but less likely to be of black or minority ethnic origin (14% vs 25%).[31]

Comparison with other Work

We have been able to present an in-depth and nuanced description of GPs' understanding of spiritual health, with a study that is larger than previous work. We identified some views of spiritual health that were similar to those expressed in interviews with 19 Scottish GPs and a quantitative survey of 87 GP trainers, including a lack of meaning and links with humanity and the divine. [27 32] The breadth of views in our study was similar, enabling us to map responses to a framework based on Appleby's four descriptors of attitudes to spiritual care. Other work has focussed on a priori definitions of spiritual health.[28 33-35] Most literature originates from the USA, which may promote a definition that is appropriate only to that socio-cultural context.

For peer review only - http://bmjopen.bmj.com/site/about/guidelines.xhtml

Implications for Research and Practice

Spiritual health proved to be a divisive topic that evoked strong opinions. The GMC and the RCGP expect GPs to include spiritual health in care, but this research exposes heterogeneity in GPs' understanding of the topic. Whether, and how, an individual doctor's understanding of spiritual health influences their practice, is unknown. It is possible that discussion of spiritual health leads to enhanced patient outcomes, in areas such as end of life care. The positive, negative, and unintended, consequences of introducing discussion of spiritual health into consultations requires further study. Busy GPs are likely to require evidence of benefit before they would routinely widen the scope of their existing practice. Impact on workload is an important area for future scrutiny. GPs are not well placed to provide spiritual care,[35] and how to ensure people with spiritual needs are directed to appropriate support requires further investigation. Social prescribing and primary care chaplains offer potential routes to spiritual support for patients,[36] and both may help to reduce demands on doctors in primary care.

CONCLUSIONS

Views on spiritual health amongst GPs are heterogeneous. A clear definition would facilitate future research, and may help to promote discussion in the consultation. The themes identified in this study are generated from working GPs and could form the basis of a socio-culturally sensitive and encompassing definition of spiritual care. Without a shared understanding, holistic assessment of patients'needs and referral for appropriate support will be an ongoing challenge.

For peer review only

For peer review only - http://bmjopen.bmj.com/site/about/guidelines.xhtml

Funding: Author 1 was funded by a post-CCT GP Fellowship funded by Health Education North East and Durham Dales and Easington Clinical Commissioning Group, and funds from the National Institute for Health Research (NIHR) School for Primary Care Research. Grant reference HEE REF 0150/8116. Author 3 was part funded by the North East and North Cumbria Applied Research Collaboration.

The views expressed are those of the author(s) and not necessarily those of DDES CCG, the NIHR or the Department of Health and Social Care.

Author's statement: OW conceived the study, with input from BH and CJ. OW developed the study design with input from CJ and BH. OW developed the survey, with input from BH and CJ. OW performed the analysis and wrote the first and successive drafts of the manuscript. OW, CJ and BH interpreted the data, critically revised the manuscript for important intellectual content, and approved the final version of the manuscript.

Ethical approval: Ethics approval was sought and obtained from Newcastle University on 27/2/2019

Consent: Participants were asked to give their informed consent before participating

Competing interests: None

Data statement: Data are saved on Newcastle University secure servers and may be available in negotiation with the first author.

Acknowledgements

Thank you to all the GPs who participated, and all those in CCGs and CRNs who helped with recruitment. Thank you to Voice North members who helped shape the study and analysis.

Box 1. Definitions of spiritual health used in research

- Humanity- spirituality, the spirit or the soul is part of the universal human experience[20 37]

For peer review only - http://bmjopen.bmj.com/site/about/guidelines.xhtml

For peer review only

- Meaning and purpose, or self-actualisation[17 35 37-40]
- Inner strength, peace, and resilience[38]
- Transcendence, or a sense of connectivity to aspects outside the self, for example with the community, society, family and nature[17 37 39 40]
- Relationship with the divine or sacred[37]
- Expression- beliefs, values, traditions, rituals and practices[20 39]

Table 1 Characteristics of respondents and self reported spirituality and religiosity

	**Number (%)** (n=177)
**Sex**	
Male	65 (37%)
Female	111 (63%)
**Ethnic Group**	
White British	139 (79%)
Any other White background,	7 (4%)
White Irish	5 (3%)
Indian	6 (3%)
Any other Mixed / Multiple ethnic background	4 (2%)
Other background	15 (9%)
**Religion**	
Christian	110 (63%)
Other	12 (7%)
None	49 (28%)

For peer review only - http://bmjopen.bmj.com/site/about/guidelines.xhtml

For peer review only

**Country of primary medical qualification**	
England	144 (81%)
Scotland	14 (8%)
Elsewhere in Europe	8 (5%)
Asia	6 (3%)
Africa or Americas	5 (3%)
**Country of GP training**	
England	168 (95%)
Scotland	5 (3%)
Other	4 (2%)
**I am a spiritual person***	
Agree	122 (69%)
Disagree	28 (16%)
No opinion	27 (15%)
**I am a religious person***	
Agree	85 (47%)
Disagree	67 (39%)
No opinion	25 (14%)
**I am spiritually healthy***	
Agree	128 (72%)
Disagree	4 (2%)
No opinion	45 (25%)

*Responses to a 5 point Likert scale, with strongly agree/disagree and agree/disagree aggregated

For peer review only - http://bmjopen.bmj.com/site/about/guidelines.xhtml

For peer review only

Table 2 Attitudes to spiritual care: GP views mapped onto a framework of enthusiasm for spiritual care

**Categories of attitudes to Spiritual Care amongst GPs identified in a quantitative survey by Appleby et al [27]**	**Example of attitude**	**Views expressed by GPs in this survey.**
**Rejecting**	Discomfort with the concept and any spiritual health care	"Not comfortable including spirituality in consultations." Male, GP for 20-30 years<br>"What can I say. There is no evidence for any God." Male, no religion, GP for 20-30 years<br>"I don't feel this has anything to do with my role as a GP." Female, no religion, GP for 6-10 years<br>"I'm not sure we need to be the people to ask about spiritual things. This sits better with a religious leader. I don't feel asking these questions really helps me to help them. It doesn't sit comfortably with a GP role in my opinion." Female, Christian, GP for 20-30 years
**Guarded**	Limited circumstances where a GP should be involved	"I think I am very hesitant in asking as you hear of doctors being accused of causing offence etc." Female, Christian, GP for 6-10 years<br>"I don't see my role to take over a spiritual conversation unless directly relevant to medical choices." Female, no religion, GP for 20-30 years<br>"I'm not sure this is my place and the only discussion I might have would be at the end of life." Female, no religion, GP for 6-10 years<br>"Signposting to appropriate people is appropriate and probably a better use of the patient's time." Male, no religion, GP for 6-10 years<br>"Unless patient brings it up or it's an end of life planning content it's none of my business." Female, Christian, GP for 6-10 years
**Pragmatic**	Willing to address the topic at patient request	"I can remember the first time I asked a palliative patient about whether they had anyone they discussed spiritual matters with, I was nervous about asking, but the

For peer review only

		amazing response has made me ask almost all of my palliative patients since." Male, no religion, GP for 11-20 years<br>"I will NEVER bring it up but would engage professionally with the patient in a positive manner." Male, no religion, GP for 20-30 years<br>"I feel comfortable discussing spirituality if led by the patient." Female, Christian, GP for 0-5 years<br>"The apparent importance of the spiritual aspects in Derek's life and pending death would encourage to broach this more explicitly and respond accordingly." Male, Christian, GP for 20-30 years
**Embracing**	Enthusiasm for the topic, and acceptance of the importance in healthcare	"By comfortable I mean that I'm not afraid to discuss these things and think it's important." Female, Christian, GP for 11-20 years<br>"I would be comfortable asking what he is reading in the bible and discussing passages that may bring comfort to him and his wife if they wanted." Female, Christian, GP for 20-30 years<br>"They have hugely valued talking about faith and praying together as we share the same faith. I have been able to still explore faith with those of different faiths." Male, Christian, GP for 11-12 years

For peer review only

For peer review only - http://bmjopen.bmj.com/site/about/guidelines.xhtml

REFERENCES

1. Mark RC, Christina MP, Bruce R. Oxford Textbook of Spirituality in Healthcare. Oxford, UK.
2. National Health Service. 5 steps to mental wellbeing 2018 [Available from: https://www.nhs.uk/conditions/stress-anxiety-depression/improve-mental-wellbeing/ accessed 24/7/19.
3. Royal College of General Practitioners. The RCGP Curriculum: Professional & Clinical Modules. In: Practitioners RCoG, ed. London, 2016.
4. General Medical Council. Good Medical Practice In: Council GM, ed. London, 2013.
5. National Institute of Health and Care Excellence. Care of dying adults in the last days of life. NICE guideline NG31, 2015.
6. Yang GM, Tan YY, Cheung YB, et al. Effect of a spiritual care training program for staff on patient outcomes. *Palliative and Supportive Care* 2016;15(4):434-43. doi: 10.1017/S1478951516000894 [published Online First: 11/29]
7. Brémault-Phillips S, Olson J, Brett-MacLean P, et al. Integrating spirituality as a key component of patient care. *Religions* 2015;6(2):476-98. doi: 10.3390/rel6020476
8. Zimmer Z, Jagger C, Chiu C-T, et al. Spirituality, religiosity, aging and health in global perspective: A review. *SSM - Population Health* 2016;2:373-81. doi: https://doi.org/10.1016/j.ssmph.2016.04.009
9. Powell LH, Shahabi L, Thoresen CE. Religion and spirituality: Linkages to physical health. *American psychologist* 2003;58(1):36.
10. Koenig H. Religion, Spirituality, and Health: The Research and Clinical Implications2012.
11. Rew L, Wong YJ. A systematic review of associations among religiosity/spirituality and adolescent health attitudes and behaviors. *Journal of adolescent health* 2006;38(4):433-42.
12. Bornet M-A, Edelmann N, Rochat E, et al. Spiritual care is stagnating in general practice: the need to move towards an embedded model. *British Journal of General Practice* 2019;69(678):40. doi: 10.3399/bjgp19X700613
13. Poole R, Cook CCH. Praying with a patient constitutes a breach of professional boundaries in psychiatric practice. *British Journal of Psychiatry* 2018;199(2):94-98. doi: 10.1192/bjp.bp.111.096529 [published Online First: 01/02]
14. Bentley C. Christian doctor faces new investigation over prayer with patients 2019 [Available from: https://www.premier.org.uk/News/UK/Christian-doctor-faces-new-investigation-over-prayer-with-patients accessed 4/8/19.
15. Tanyi RA, McKenzie M, Chapek C. How family practice physicians, nurse practitioners, and physician assistants incorporate spiritual care in practice. *Journal of the American Academy of Nurse Practitioners* 2009;21(12):690-97. doi: 10.1111/j.1745-7599.2009.00459.x
16. Reinert KG, Koenig HG. Re-examining definitions of spirituality in nursing research. *Journal of Advanced Nursing* 2013;69(12):2622-34. doi: 10.1111/jan.12152
17. Rumbold BD. A Review of Spiritual Assessment in Health Care Practice. *The Medical Journal of Australia* 2007;186(10):s60-62.
18. Assing Hvidt E, Sondergaard J, Ammentorp J, et al. The existential dimension in general practice: identifying understandings and experiences of general practitioners in Denmark. *Scandinavian Journal of Primary Health Care* 2016;34(4):385-93.

For peer review only - http://bmjopen.bmj.com/site/about/guidelines.xhtml

For peer review only

19. Holmes SM, Rabow MW, Dibble SL. Screening the soul: communication regarding spiritual concerns among primary care physicians and seriously ill patients approaching the end of life. *American Journal of Hospice & Palliative Medicine* 2006;23(1):25-33.
20. Saguil A, Phelps K. The spiritual assessment. *American Family Physician* 2012;86(6):546-50.
21. Monroe MH, Bynum D, Susi B, et al. Primary care physician preferences regarding spiritual behavior in medical practice. *Archives of Internal Medicine* 2003;163(22):2751-6.
22. McCauley J, Jenckes MW, Tarpley MJ, et al. Spiritual beliefs and barriers among managed care practitioners. *Journal of Religion & Health* 2005;44(2):137-46.
23. Larimore WL. Providing basic spiritual care for patients: Should it be the exclusive domain of pastoral professionals? *American Family Physician* 2001;63(1):36-40.
24. Daaleman TP, Nease DE, Jr. Patient attitudes regarding physician inquiry into spiritual and religious issues. *Journal of Family Practice* 1994;39(6):564-8.
25. Brennan M, Heiser D. Introduction: Spiritual Assessment and Intervention: Current Directions and Applications. *Journal of Religion, Spirituality & Aging* 2004;17(1-2):1-20. doi: 10.1300/J496v17n01_01
26. Lee-Poy M, Stewart M, Ryan BL, et al. Asking patients about their religious and spiritual beliefs: Cross-sectional study of family physicians. *Canadian Family Physician*;62(9):e555-61.
27. Appleby A, Swinton J, Bradbury I, et al. GPs and spiritual care: signed up or souled out? A quantitative analysis of GP trainers' understanding and application of the concept of spirituality. *Education for Primary Care* 2018;29(6):367-75.
28. Best M, Butow P, Olver I. Do patients want doctors to talk about spirituality? A systematic literature review. *Patient Education and Counseling* 2015;98(11):1320-28. doi: https://doi.org/10.1016/j.pec.2015.04.017
29. Puchalski CM, Vitillo R, Hull SK, et al. Improving the spiritual dimension of whole person care: reaching national and international consensus. *Journal of palliative medicine* 2014;17(6):642-56. doi: 10.1089/jpm.2014.9427
30. Guest G, MacQueen KM, Namey EE. Applied thematic analysis 'Themes and Codes' chapter. Thousand Oaks, CA: SAGE 2012.
31. The General Medical Council. What Our Data Tells us about General Practitioners working for the NHS in England and Scotland, 2018.
32. Appleby A, Swinton J, Wilson P. What GPs mean by 'spirituality' and how they apply this concept with patients: a qualitative study. *BJGP open* 2018; 2(2). (accessed 2018/07//).
33. Borneman T, Ferrell B, Puchalski CM. Evaluation of the FICA Tool for Spiritual Assessment. *Journal of Pain and Symptom Management* 2010;40(2):163-73. doi: 10.1016/j.jpainsymman.2009.12.019
34. Lucchetti G, Bassi RM, Lucchetti ALG. Taking Spiritual History in Clinical Practice: A Systematic Review of Instruments. *EXPLORE: The Journal of Science and Healing* 2013;9(3):159-70. doi: https://doi.org/10.1016/j.explore.2013.02.004
35. Puchalski CM. Integrating spirituality into patient care: an essential element of person‐centered care. *Polskie Archiwum Medycyny Wewnetrznej* 2013;123(9):491-97.
36. Brandling J, House W. Social prescribing in general practice: adding meaning to medicine. *British Journal of General Practice* 2009;59(563):454. doi: 10.3399/bjgp09X421085
37. Puchalski CM. The FICA Spiritual History Tool #274. *Journal of Palliative Medicine* 2014;17(1):105-6.
38. Paal P, Helo Y, Frick E. Spiritual Care Training Provided to Healthcare Professionals: A Systematic Review. *Journal of Pastoral Care & Counseling* 2015;69(1):19-30. doi: 10.1177/1542305015572955
39. Mueller PS, Plevak DJ, Rummans TA. Religious involvement, spirituality, and medicine: implications for clinical practice. *Mayo Clinic Proceedings* 2001;76(12):1225-35.
40. Larimore WL, Parker M, Crowther M. Should clinicians incorporate positive spirituality into their practices? What does the evidence say? *Annals of Behavioral Medicine* 2002;24(1):69-73.

For peer review only - http://bmjopen.bmj.com/site/about/guidelines.xhtml

For peer review only

For peer review only - http://bmjopen.bmj.com/site/about/guidelines.xhtml

Standards for Reporting Qualitative Research (SRQR)*

http://www.equator-network.org/reporting-guidelines/srqr/

Title and abstract

	Page/line no(s).
**Title** - Concise description of the nature and topic of the study Identifying the study as qualitative or indicating the approach (e.g., ethnography, grounded theory) or data collection methods (e.g., interview, focus group) is recommended	0
**Abstract** - Summary of key elements of the study using the abstract format of the intended publication; typically includes background, purpose, methods, results, and conclusions	0

Introduction

**Problem formulation** - Description and significance of the problem/phenomenon studied; review of relevant theory and empirical work; problem statement	2, 3
**Purpose or research question** - Purpose of the study and specific objectives or questions	3

Methods

**Qualitative approach and research paradigm** - Qualitative approach (e.g., ethnography, grounded theory, case study, phenomenology, narrative research) and guiding theory if appropriate; identifying the research paradigm (e.g., postpositivist, constructivist/ interpretivist) is also recommended; rationale**	3, 4
**Researcher characteristics and reflexivity** - Researchers' characteristics that may influence the research, including personal attributes, qualifications/experience, relationship with participants, assumptions, and/or presuppositions; potential or actual interaction between researchers' characteristics and the research questions, approach, methods, results, and/or transferability	4
**Context** - Setting/site and salient contextual factors; rationale**	2,3
**Sampling strategy** - How and why research participants, documents, or events were selected; criteria for deciding when no further sampling was necessary (e.g., sampling saturation); rationale**	1, 4
**Ethical issues pertaining to human subjects** - Documentation of approval by an appropriate ethics review board and participant consent, or explanation for lack thereof; other confidentiality and data security issues	10
**Data collection methods** - Types of data collected; details of data collection procedures including (as appropriate) start and stop dates of data collection and analysis, iterative process, triangulation of sources/methods, and modification of procedures in response to evolving study findings; rationale**	3, 4

For peer review only - http://bmjopen.bmj.com/site/about/guidelines.xhtml

For peer review only

**Data collection instruments and technologies** - Description of instruments (e.g., interview guides, questionnaires) and devices (e.g., audio recorders) used for data collection; if/how the instrument(s) changed over the course of the study	3
**Units of study** - Number and relevant characteristics of participants, documents, or events included in the study; level of participation (could be reported in results)	4 (table 1)
**Data processing** - Methods for processing data prior to and during analysis, including transcription, data entry, data management and security, verification of data integrity, data coding, and anonymization/de-identification of excerpts	3, 4
**Data analysis** - Process by which inferences, themes, etc., were identified and developed, including the researchers involved in data analysis; usually references a specific paradigm or approach; rationale**	3,4
**Techniques to enhance trustworthiness** - Techniques to enhance trustworthiness and credibility of data analysis (e.g., member checking, audit trail, triangulation); rationale**	4, 5, 6, 7 (table 2)

Results/findings

**Synthesis and interpretation** - Main findings (e.g., interpretations, inferences, and themes); might include development of a theory or model, or integration with prior research or theory	5, 6, 7 (table 2)
**Links to empirical data** - Evidence (e.g., quotes, field notes, text excerpts, photographs) to substantiate analytic findings	4, 5, 6, 7, (table 2)

Discussion

**Integration with prior work, implications, transferability, and contribution(s) to the field -** Short summary of main findings; explanation of how findings and conclusions connect to, support, elaborate on, or challenge conclusions of earlier scholarship; discussion of scope of application/generalizability; identification of unique contribution(s) to scholarship in a discipline or field	8
**Limitations** - Trustworthiness and limitations of findings	1

Other

**Conflicts of interest** - Potential sources of influence or perceived influence on study conduct and conclusions; how these were managed	10
**Funding** - Sources of funding and other support; role of funders in data collection, interpretation, and reporting	10

*The authors created the SRQR by searching the literature to identify guidelines, reporting standards, and critical appraisal criteria for qualitative research; reviewing the reference lists of retrieved sources; and contacting experts to gain feedback. The SRQR aims to improve the transparency of all aspects of qualitative research by providing clear standards for reporting qualitative research.

For peer review only - http://bmjopen.bmj.com/site/about/guidelines.xhtml

**The rationale should briefly discuss the justification for choosing that theory, approach, method, or technique rather than other options available, the assumptions and limitations implicit in those choices, and how those choices influence study conclusions and transferability. As appropriate, the rationale for several items might be discussed together.

Reference:

O'Brien BC, Harris IB, Beckman TJ, Reed DA, Cook DA. **Standards for reporting qualitative research: a synthesis of recommendations.** *Academic Medicine*, Vol. 89, No. 9 / Sept 2014 DOI: 10.1097/ACM.0000000000000388

For peer review only

For peer review only - http://bmjopen.bmj.com/site/about/guidelines.xhtml

BMJ Open

What Do Doctors Understand By 'Spiritual Health'? A survey of UK general practitioners

Journal:	*BMJ Open*
Manuscript ID	bmjopen-2020-045110.R1
Article Type:	Original research
Date Submitted by the Author:	04-May-2021
Complete List of Authors:	Whitehead, Orla; Newcastle University, Population Health Sciences Institute<br>Jagger, Carol; Newcastle University, Population Health Sciences Institute<br>Hanratty, Barbara; Newcastle University, Population Health Sciences Institute
**Primary Subject Heading**:	General practice / Family practice
Secondary Subject Heading:	Qualitative research
Keywords:	PRIMARY CARE, PALLIATIVE CARE, MENTAL HEALTH

SCHOLARONE™
Manuscripts

I, the Submitting Author has the right to grant and does grant on behalf of all authors of the Work (as defined in the below author licence), an exclusive licence and/or a non-exclusive licence for contributions from authors who are: i) UK Crown employees; ii) where BMJ has agreed a CC-BY licence shall apply, and/or iii) in accordance with the terms applicable for US Federal Government officers or employees acting as part of their official duties; on a worldwide, perpetual, irrevocable, royalty-free basis to BMJ Publishing Group Ltd ("BMJ") its licensees and where the relevant Journal is co-owned by BMJ to the co-owners of the Journal, to publish the Work in this journal and any other BMJ products and to exploit all rights, as set out in our licence.

The Submitting Author accepts and understands that any supply made under these terms is made by BMJ to the Submitting Author unless you are acting as an employee on behalf of your employer or a postgraduate student of an affiliated institution which is paying any applicable article publishing charge ("APC") for Open Access articles. Where the Submitting Author wishes to make the Work available on an Open Access basis (and intends to pay the relevant APC), the terms of reuse of such Open Access shall be governed by a Creative Commons licence – details of these licences and which Creative Commons licence will apply to this Work are set out in our licence referred to above.

Other than as permitted in any relevant BMJ Author's Self Archiving Policies, I confirm this Work has not been accepted for publication elsewhere, is not being considered for publication elsewhere and does not duplicate material already published. I confirm all authors consent to publication of this Work and authorise the granting of this licence.

For peer review only

TITLE: What do doctors understand by spiritual health? A survey of UK general practitioners

Authors : Orla Whitehead Population Health Sciences Institute Faculty of Medical Sciences Newcastle University, Carol Jagger Population Health Sciences Institute Faculty of Medical Sciences Newcastle University, Barbara Hanratty Population Health Sciences Institute Faculty of Medical Sciences Newcastle University

orla.whitehead@newcastle.ac.uk, carol.jagger@newcastle.ac.uk, barbara.hanratty@newcastle.ac.uk

Keywords: Spirituality, General Practitioners, Consensus, United Kingdom, Religion, Spiritual Therapies, Surveys and Questionnaires, Referral and Consultation, Spiritual Health.

Word count: 2890

Corresponding author: Dr Orla Whitehead, Newcastle University, Biomedical Research Building, Campus for Ageing and Vitality, Newcastle upon Tyne, NE4 5PL orla.whitehead@newcastle.ac.uk

ABSTRACT

Background

In the UK, doctors' regulatory and professional bodies require general practitioners to consider discussing spiritual health as part of the consultation. However, spiritual health is not defined in guidance, and it is unknown what individual doctors understand by the term.

Research question:

What do GPs understand by the term 'spiritual health'?

Aim

To explore how GPs understand and define spiritual health.

Design and setting

Survey of GPs in England 9th April-21st May 2019

For peer review only - http://bmjopen.bmj.com/site/about/guidelines.xhtml

Method

A mixed methods online survey asked practicing GPs in England qualitative free text questions- “What does the term 'Spiritual Health' mean to you?” and “Any comments?” after five vignettes about discussing spiritual health with patients. These were subject to thematic analysis using a priori themes from the literature on GP definitions of spiritual health, and on attitudes towards the topic.

Participants

177 practicing GPs in England

Results

177 GPs responded to the survey. Understanding of spiritual health fitted into three themes: self-actualisation and meaning, transcendence and relationships beyond the self, and expressions of spirituality. A full range of views were expressed, from a minority who challenged their role in spiritual health, through to others enthusiastic about its place in health care.

Conclusion

Spirituality and religiosity are understood by English GPs to be distinct concepts.. A consensus definition of spiritual health incorporating the themes identified by working doctors, may be helpful to support GPs to follow the recommended guidance in their practice.

ARTICLE SUMMARY

Strengths and Limitations

- Spiritual health is an undefined concept in guidance for GPs, but UK doctors have it within their professional remit. This survey offers a threefold definition of spiritual health developed by family doctors.
- This is the largest survey of doctors in Europe on the topic of spiritual health, as 177 practicing GPs responded, a large number from a group who are often difficult to engage in research. A broad range of views was expressed, suggesting that selection bias was minimal.

For peer review only

- Data were collected on religious views, despite this being a potentially sensitive topic.

For peer review only

For peer review only - http://bmjopen.bmj.com/site/about/guidelines.xhtml

INTRODUCTION

Modern medicine may have little time for the spiritual aspects of life, however, doctors have always been required to address more than physical problems.[1] Within the NHS, interest in holistic care and mental wellbeing is growing.[2] Holism underpins primary care, and general practitioners (GPs) are expected to treat patients within their social and cultural context.[3] The UK regulatory body, the General Medical Council (GMC), expects doctors to consider the religious, social and cultural aspects of a patient's problem,[4] and the Royal College of General Practitioners (RCGP) includes spiritual health in their curriculum for GP trainees.[3] National Institute for Health and Care Excellence guidance also recommends that discussion of spiritual beliefs should be part of the care of dying patients.[5] A recent article has advocated for an 'embedded' model of spiritual care within UK general practice.[6] Discussing spiritual health may be a beneficial intervention for patients,[7] and there has been speculation that it could increase job satisfaction for GPs.[8] Spirituality can also be a resource for health and wellbeing. Religiosity and spirituality are associated with longevity[9 10], and unmet spiritual needs may be detrimental to health.[11] Research into interactions between spirituality and health is a growing field.[9 12] In recent years, arguments have been made for the importance of embedding spiritual health within general practice.[6] However, the interface between spirituality and medicine can be controversial.[13] Pathological spiritual and religious beliefs may develop, and some UK GPs who shared their faith with patients have been investigated by the GMC.[14]

There is no consensus or widely accepted definition of spiritual health, despite its proposed place in GPs' work. Spirituality means different things to different people, and understanding may shift across the lifecourse.[15] Some definitions invoke the symptomatology of mental illness, which causes confusion and negative attitudes to the concept.[16 17] Danish authors have argued that the term 'existential health' is more appropriate for a secular multicultural society,[18] while others see existential and spiritual health as synonymous.[19] Similarly, spirituality and religiosity are often conflated.[20-24] For some, spirituality is personality trait predisposing to concern with the metaphysical[25] that has played an important role in human societies and medicine.[26] Previous quantitative survey work with GP trainers in Scotland found that spirituality was perceived by educators as a meaningful, useful, but unclear concept.[27] Researchers investigating how spiritual health should be discussed in primary care have adopted a variety of definitions drawing themes from traditional religion

For peer review only - http://bmjopen.bmj.com/site/about/guidelines.xhtml

through to humanism (Box 1). One of the most frequently adopted is a consensus definition from international holistic care conferences that encompasses all the concepts listed in Box 1, apart from inner peace, strength and resilience. [27 28 29] However, while these definitions may be useful to those interested in the topic, or within academia, it is not known what working definition is being used by GPs in a clinical context.

A clear understanding of spiritual health will be essential if it is to become a routine part of holistic care in general practice. This study aims to explore how GPs understand and define spiritual health in their day to day practice, and build the evidence base regarding GP attitudes towards the topic.

Insert Box 1 here (Definitions of spiritual health used in research)

METHOD

Design

A mixed methods online survey was developed and used to explore GP definitions of the term 'spiritual health', attitudes towards the topic, and views on discussing spiritual health with their patients. (Data on the use of structured tools to support discussions within consultations are reported elsewhere). Information collected from the participants included demographic characteristics (sex, ethnicity and religion); geographical region and years of work experience. Participants were asked to rate themselves on a five point Likert scale, in response to two statements: 1. I am a spiritual person and 2. I am a religious person. Spiritual health was deliberately undefined in the survey; GPs were asked 'what does the term spiritual health mean to you?' and invited to share any other thoughts on this topic, prompted by five patient vignettes.

The survey was sent to qualified GPs in England who were working in standard general practice settings, out of hours, and urgent care. Included were all practicing GPs in England, people who were temporarily out of practice (for example maternity leave) were encouraged to participate, but those who had retired and would not return to practice were excluded. The survey was sent via Clinical Commissioning Group (CCG) lists, CCG newsletters, professional groups, and practice managers in Spring/Summer 2019, all 211 CCGs were approached, both convenience and snowball sampling methods were used.

For peer review only - http://bmjopen.bmj.com/site/about/guidelines.xhtml

Data Analysis

Free text responses to the question 'what does the term spiritual health mean to you', were analysed using a deductive thematic analysis, based upon a priori themes developed by immersion in the literature (Box 1). The analysis process followed a standard path: reading and rereading of the data, coding based upon key words and ideas expressed by respondents, comparing and contrasting of similar codes, aggregation into topics that were then grouped into the a priori themes, and any other additional themes.[30] These themes were reviewed to develop the central concepts within the data. Outlying cases were examined, to identify insights from respondents most and least comfortable with the topic. Qualitative software was not used to manage these data, rather the concepts were manipulated using coloured pens, paper and scissors. The researchers were cognisant of their own identities, cultures and religious backgrounds in their approach to the topic. Feedback was sought from GPs from other backgrounds in the survey development. Authors 1 and 3 are practicing GPs.

A similar process was followed to analyse the free text provided in the space for additional comments. In this case, GP responses were also mapped against the attitudes to spiritual care identified in Appleby and colleagues' quantitative work with Scottish GP trainers. They identified four categories of attitudes labelled as rejecting, guarded, pragmatic and embracing.[27]

Patient and Public Involvement

Patient and Public Involvement was important to the development of the research and analysis, and the authors wish to acknowledge, and are grateful for, the contribution of Voice North. The group had diverse opinions on the topic, but all recognised the need for holistic care, and the utility of a working definition of 'spiritual health'.

RESULTS

177 practicing English GPs responded to the survey, shown in table 1. The majority were female, of white British origin, and with a primary medical qualification obtained in the UK. Most respondents (69%) considered themselves to be spiritual people. Just under half (48%) considered themselves religious, 70% of respondents reported that they had a religion (63%

For peer review only - http://bmjopen.bmj.com/site/about/guidelines.xhtml

Christian, 7% named other religions). One fifth of respondents (21%) reported that they were spiritual, not religious. All respondents who agreed they were religious also agreed they were spiritual people.

Insert Table 1 here (Characteristics of respondents and self reported spirituality and religiosity)

157 out of 177 participants provided a free text response to the question 'what does the term spiritual health' mean to you?' These responses, ranging in length from 1-105 words, were subject to a thematic analysis. Most of the definitions and understanding of spiritual health could be coded into the three a priori themes: 1) self-actualisation and meaning, 2) transcendence and relationships beyond the self, and 3) expressions of spirituality. One additional theme was developed, and labelled 4) 'meaningless'. Religious participation cut across all three of these themes; development of the self is included in many world religions, as is transcendence, community and relationships to other humans, the world, and/or the divine, and of course expression of beliefs. Only one respondent used the term existential health, and a small number of respondents commented that spiritual health is synonymous with mental health. In the description of themes below, direct quotations from participants are included within the text without attribution.

Self-Actualisation and Meaning

Self-actualisation and meaning brought together ideas around humanity/human experience, a sense of spirit or soul, inner strength, peace and resilience and meaning, purpose or self-actualisation. Acceptance of the self, the situation, with a sense of peace and meaning to life was mentioned, both with and without reference to God. Participants referred to the health of a soul, or inner self, which could be either peaceful or disrupted with concerns, conflict, or guilt. A sense of purpose was viewed as giving fulfilment and meaning to life, or as an understanding of the identity: "Understanding where I have come from and where I am heading." Personal resilience and inner strength were mentioned as a healthy outcome of acceptance of the soul, of our humanity, and of our status as 'finite beings.' One participant mentioned "security in who we are, and why we're here", expressing that sense of personal identity and purpose that appears to be part of the human experience. Looking into the self, and something of the individual that is "neither physical nor mental", and finding peace and

For peer review only - http://bmjopen.bmj.com/site/about/guidelines.xhtml

meaning there, is core to this concept. However, few participants mentioned ideas without going on to refer to the position of the individual and their self-actualisation within the world/universe, “understanding your own place and role within society.”

Transcendence and Relationship beyond the Self

This theme encompassed ideas of connections to community, nature and family, with relationship with the divine and sacred. A majority of participants mentioned relationship and connections to the community, ‘something bigger’, family, and the world/nature, “Being in tune with people. Enjoying and respecting the natural world.” Participants referred to: “believing in something bigger than just you,” and “connectedness to the world they can’t see.” Participants were often keen to be inclusive in their definitions, referring to “being comfortable with the world around you and any faith entity you believe in”, or referring to “a higher being” rather than a specific deity. Some participants were clear that, for them, this was part of an accepted religious framework, for example “a relationship with Jesus” (one participant) or “myself as a Child of God” (18 participants mentioning God).”. Looking outwards, “the state of one’s capacity for love” and a belonging to something outside the self, appears to underpin this concept.

Expression of Spirituality

The concepts of spirituality of the self, and of our connections outside the self, come together into expressions of spirituality, such as values, rituals, practices, and lifestyles. One participant stated “it embraces everything from mindfulness to religious practice (short of pathological religious delusion.)” Many participants mentioned having ‘faith’, although what this was faith in was not always expanded upon. The freedom to be “able and …visibly practice my faith” was mentioned by one participant, suggesting that spiritual health requires freedom of expression to be permitted. The use of morals, and personal ethics to influence behaviour, for example “believing in something abstract that guides behaviour and thinking”, was mentioned as part of spiritual health. The expression of these values and morals was often linked to membership and belonging to a community, or the seeking of self-actualisation.

Spiritual Health as a Meaningless term

A small minority of respondents (6%) reported that spiritual health conveyed no meaning, or was unclear, to them, but there was little elaboration on this assertion.

For peer review only - http://bmjopen.bmj.com/site/about/guidelines.xhtml

For peer review only

Attitudes to spiritual care

Respondents were invited to add any other thoughts on spiritual health and care in general through unprompted free text boxes asking "any comments". A third of participants contributed their views, and data relating to understanding and definition of spiritual health were mapped to an existing framework developed by Appleby and colleagues in Table 2. This is derived from a small qualitative survey of 19 Scottish GPs[31] which was then used by the authors with a larger group of Scottish GPs in a quantitative study[27], and presents a categorisation of the spectrum of attitudes to spiritual care already used in a UK context. The perceived need for cues by a large number of respondents, and the low numbers of respondents who fit into the 'rejecting' attitude, would suggest that most respondents fall in to the "guarded" and/or "pragmatic" attitudes identified.

Table 2 here

Attitudes to spiritual care: GP views mapped onto a framework of enthusiasm for spiritual care

For peer review only - http://bmjopen.bmj.com/site/about/guidelines.xhtml

For peer review only

DISCUSSION

This large study of practicing English GPs suggests that spiritual health has a meaning for many doctors, but that this meaning is distinct from religion. Three themes were identified to encompass GPs' understanding of spiritual health: self-actualisation and meaning, transcendence and relationships beyond the self, and expression of spirituality. Difficulty in articulating exactly what is conveyed by the term spiritual health was a common challenge.

Comparison with other Work

We have been able to present an in-depth and nuanced description of GPs' understanding of spiritual health, with a study that is larger than previous work in the UK. We identified some views of spiritual health that were similar to those expressed in interviews with 19 Scottish GPs and a quantitative survey of 87 GP trainers, including a lack of meaning of the term spiritual, links with humanity and the divine. [27 31]The breadth of views in our study was similar, enabling us to map responses to a framework based on Appleby's four descriptors of attitudes to spiritual care, as shown in table 2. Other work has focussed on a priori definitions of spiritual health,[28 32-34] and while Puchalski et al's consensus definition[29] gives a useful expert definition, it was previously unknown what definition English GPs were utilising in their work. The definitions given by English GPs reflects those given in the literature (box 1). While some authors describe religion and spirituality as interchangeable[35], a fifth of the GP participants here described themselves as spiritual but not as religious, showing they believe these are different concepts. Most other literature originates from the USA, which may promote a definition that is appropriate only to that socio-cultural context, whereas building on Appleby et al allows the UK evidence base to be developed. Assing Hvidt et al's work on 'existential health' in Denmark[36] is an interesting comparator to this work, however their definition of existential health differs from that of 'spiritual health' here by English GPs, possibly due to cultural differences between the UK and Denmark.

Limitations

While the response rate was good for this sort of survey, it was a small proportion of GPs in England, and there could be sampling bias with those with an interest in the topic being more likely to respond. It is not known whether the high rate of identification as a "spiritual person" is due to this being the case in the wider GP population, or just those sampled. Unfortunately, the sample was not representative of GPs with non-white backgrounds.

For peer review only - http://bmjopen.bmj.com/site/about/guidelines.xhtml

Respondents differed from the UK GP population registered with the GMC, which should be considered alongside the findings. Compared to the GP population registered with the GMC, respondents were more likely to be female (63% vs 53%) and to have a medical qualification from the UK (89% vs 79%), but less likely to be of black or minority ethnic origin (14% vs 25%).[37]

Recruiting GPs via CCG newsletters and other forms of publicity resulted in an good sample size, but provides no information on people who decided not to take part. The range of views that were expressed provides some confidence in our approach, but we acknowledge that people with strong views from either the “rejecting” or “embracing” ends of the spectrum may be more likely to have participated. Respondents were drawn from across England, with a majority in the north (63%). This was probably due to greater engagement with the project from CCGs in the area local to the research team.

Respondents differed from the UK GP population registered with the GMC, which should be considered alongside the findings. Compared to the GP population registered with the GMC, respondents were more likely to be female (63% vs 53%) and to have a medical qualification from the UK (89% vs 79%), but less likely to be of black or minority ethnic origin (14% vs 25%).[37]

Implications for Research and Practice

Spiritual health proved to be a divisive topic that evoked strong opinions. The GMC and the RCGP expect GPs to include spiritual health in care, but this research exposes heterogeneity in GPs’ understanding of the topic. Whether, and how, an individual doctor’s understanding of spiritual health influences their practice, is unknown. It is possible that discussion of spiritual health leads to enhanced patient outcomes, in areas such as end of life care. The positive, negative, and unintended, consequences of introducing discussion of spiritual health into consultations requires further study. Busy GPs are likely to require evidence of benefit before they would routinely widen the scope of their existing practice. Impact on workload is an important area for future scrutiny. GPs are not well placed to provide spiritual care,[34] and how to ensure people with spiritual needs are directed to appropriate support requires further investigation. Social prescribing and primary care chaplains offer potential routes to spiritual support for patients,[38] and both may help to reduce demands on doctors in primary care.

For peer review only

For peer review only - http://bmjopen.bmj.com/site/about/guidelines.xhtml

CONCLUSIONS

Views on spiritual health amongst GPs are heterogeneous. A clear definition would facilitate future research, and may help to promote discussion in the consultation. The themes identified in this study are generated from working GPs and could form the basis of a socio-culturally sensitive and encompassing definition of spiritual care. Without a shared understanding, holistic assessment of patients'needs and referral for appropriate support will be an ongoing challenge.

For peer review only

For peer review only - http://bmjopen.bmj.com/site/about/guidelines.xhtml

Funding: Author 1 was funded by a post-CCT GP Fellowship funded by Health Education North East and Durham Dales and Easington Clinical Commissioning Group, and funds from the National Institute for Health Research (NIHR) School for Primary Care Research. Grant reference HEE REF 0150/8116. Author 3 was part funded by the North East and North Cumbria Applied Research Collaboration.

The views expressed are those of the author(s) and not necessarily those of DDES CCG, the NIHR or the Department of Health and Social Care.

Author's statement: OW conceived the study, with input from BH and CJ. OW developed the study design with input from CJ and BH. OW developed the survey, with input from BH and CJ. OW performed the analysis and wrote the first and successive drafts of the manuscript. OW, CJ and BH interpreted the data, critically revised the manuscript for important intellectual content, and approved the final version of the manuscript.

Ethical approval: Ethics approval was sought and obtained from Newcastle University on 27/2/2019

Consent: Participants were asked to give their informed consent before participating

Competing interests: None

Data statement: Data are saved on Newcastle University secure servers and may be available in negotiation with the first author.

Acknowledgements

Thank you to all the GPs who participated, and all those in CCGs and CRNs who helped with recruitment. Thank you to Voice North members who helped shape the study and analysis.

Box 1. Definitions of spiritual health used in research

- Humanity- spirituality, the spirit or the soul is part of the universal human experience[20 39]

For peer review only - http://bmjopen.bmj.com/site/about/guidelines.xhtml

- Meaning and purpose, or self-actualisation[17 34 39-42]
- Inner strength, peace, and resilience[40]
- Transcendence, or a sense of connectivity to aspects outside the self, for example with the community, society, family and nature[17 39 41 42]
- Relationship with the divine or sacred[39]
- Expression- beliefs, values, traditions, rituals and practices[20 41]

Table 1 Characteristics of respondents and self reported spirituality and religiosity

	**Number (%)** (n=177)
**Sex**	
Male	65 (37%)
Female	111 (63%)
**Ethnic Group**	
White British	139 (79%)
Any other White background,	7 (4%)
White Irish	5 (3%)
Indian	6 (3%)
Any other Mixed / Multiple ethnic background	4 (2%)
Other background	15 (9%)
**Religion**	
Christian	110 (63%)
Other	12 (7%)
None	49 (28%)

For peer review only - http://bmjopen.bmj.com/site/about/guidelines.xhtml

**Country of primary medical qualification**	
England	144 (81%)
Scotland	14 (8%)
Elsewhere in Europe	8 (5%)
Asia	6 (3%)
Africa or Americas	5 (3%)
**Country of GP training**	
England	168 (95%)
Scotland	5 (3%)
Other	4 (2%)
**I am a spiritual person***	
Agree	122 (69%)
Disagree	28 (16%)
No opinion	27 (15%)
**I am a religious person***	
Agree	85 (47%)
Disagree	67 (39%)
No opinion	25 (14%)

*Responses to a 5 point Likert scale, with strongly agree/disagree and agree/disagree aggregated

Table 2 Attitudes to spiritual care: GP views mapped onto a framework of enthusiasm for spiritual care

For peer review only - http://bmjopen.bmj.com/site/about/guidelines.xhtml

Categories of attitudes to Spiritual Care amongst GPs identified in a quantitative survey by Appleby et al [27]	Example of attitude	Views expressed by GPs in this survey.
**Rejecting**	Discomfort with the concept and any spiritual health care	"Not comfortable including spirituality in consultations." Male, GP for 20-30 years<br>"What can I say. There is no evidence for any God." Male, no religion, GP for 20-30 years<br>"I don't feel this has anything to do with my role as a GP." Female, no religion, GP for 6-10 years<br>"I'm not sure we need to be the people to ask about spiritual things. This sits better with a religious leader. I don't feel asking these questions really helps me to help them. It doesn't sit comfortably with a GP role in my opinion." Female, Christian, GP for 20-30 years
**Guarded**	Limited circumstances where a GP should be involved	"I think I am very hesitant in asking as you hear of doctors being accused of causing offence etc." Female, Christian, GP for 6-10 years<br>"I don't see my role to take over a spiritual conversation unless directly relevant to medical choices." Female, no religion, GP for 20-30 years<br>"I'm not sure this is my place and the only discussion I might have would be at the end of life." Female, no religion, GP for 6-10 years<br>"Signposting to appropriate people is appropriate and probably a better use of the patient's time." Male, no religion, GP for 6-10 years<br>"Unless patient brings it up or it's an end of life planning content it's none of my business." Female, Christian, GP for 6-10 years
**Pragmatic**	Willing to address the topic at patient request	"I can remember the first time I asked a palliative patient about whether they had anyone they discussed spiritual matters with, I was nervous about asking, but the amazing response has made me ask almost all of my palliative patients since." Male, no religion, GP for 11-20 years<br>"I will NEVER bring it up but would engage professionally with the patient in a positive manner." Male, no religion, GP for 20-30 years<br>"I feel comfortable discussing spirituality if led by the patient." Female, Christian, GP for 0-5 years

For peer review only - http://bmjopen.bmj.com/site/about/guidelines.xhtml

For peer review only

		"The apparent importance of the spiritual aspects in Derek's life and pending death would encourage to broach this more explicitly and respond accordingly." Male, Christian, GP for 20-30 years
**Embracing**	Enthusiasm for the topic, and acceptance of the importance in healthcare	"By comfortable I mean that I'm not afraid to discuss these things and think it's important." Female, Christian, GP for 11-20 years<br>"I would be comfortable asking what he is reading in the bible and discussing passages that may bring comfort to him and his wife if they wanted." Female, Christian, GP for 20-30 years<br>"They have hugely valued talking about faith and praying together as we share the same faith. I have been able to still explore faith with those of different faiths." Male, Christian, GP for 11-12 years

For peer review only

For peer review only - http://bmjopen.bmj.com/site/about/guidelines.xhtml

REFERENCES

1. Cobb M, Puchalski C, Rumbold B. Oxford Textbook of Spirituality in Healthcare. Oxford, UK.
2. National Health Service. 5 steps to mental wellbeing 2018 [Available from: https://www.nhs.uk/conditions/stress-anxiety-depression/improve-mental-wellbeing/ accessed 24/7/19.
3. Royal College of General Practitioners. The RCGP Curriculum: Professional & Clinical Modules. In: Practitioners RCoG, ed. London, 2016.
4. General Medical Council. Good Medical Practice In: Council GM, ed. London, 2013.
5. National Institute of Health and Care Excellence. Care of dying adults in the last days of life. NICE guideline NG31, 2015.
6. Bornet M-A, Edelmann N, Rochat E, et al. Spiritual care is stagnating in general practice: the need to move towards an embedded model. *British Journal of General Practice* 2019;69(678):40. doi: 10.3399/bjgp19X700613
7. Yang GM, Tan YY, Cheung YB, et al. Effect of a spiritual care training program for staff on patient outcomes. *Palliative and Supportive Care* 2016;15(4):434-43. doi: 10.1017/S1478951516000894 [published Online First: 11/29]
8. Brémault-Phillips S, Olson J, Brett-MacLean P, et al. Integrating spirituality as a key component of patient care. *Religions* 2015;6(2):476-98. doi: 10.3390/rel6020476
9. Zimmer Z, Jagger C, Chiu C-T, et al. Spirituality, religiosity, aging and health in global perspective: A review. *SSM - Population Health* 2016;2:373-81. doi: https://doi.org/10.1016/j.ssmph.2016.04.009
10. Powell LH, Shahabi L, Thoresen CE. Religion and spirituality: Linkages to physical health. *American psychologist* 2003;58(1):36.
11. Koenig H. Religion, Spirituality, and Health: The Research and Clinical Implications2012.
12. Rew L, Wong YJ. A systematic review of associations among religiosity/spirituality and adolescent health attitudes and behaviors. *Journal of adolescent health* 2006;38(4):433-42.
13. Poole R, Cook CCH. Praying with a patient constitutes a breach of professional boundaries in psychiatric practice. *British Journal of Psychiatry* 2018;199(2):94-98. doi: 10.1192/bjp.bp.111.096529 [published Online First: 01/02]
14. Bentley C. Christian doctor faces new investigation over prayer with patients 2019 [Available from: https://www.premier.org.uk/News/UK/Christian-doctor-faces-new-investigation-over-prayer-with-patients accessed 4/8/19.
15. Tanyi RA, McKenzie M, Chapek C. How family practice physicians, nurse practitioners, and physician assistants incorporate spiritual care in practice. *Journal of the American Academy of Nurse Practitioners* 2009;21(12):690-97. doi: 10.1111/j.1745-7599.2009.00459.x
16. Reinert KG, Koenig HG. Re-examining definitions of spirituality in nursing research. *Journal of Advanced Nursing* 2013;69(12):2622-34. doi: 10.1111/jan.12152
17. Rumbold BD. A Review of Spiritual Assessment in Health Care Practice. *The Medical Journal of Australia* 2007;186(10):s60-62.
18. Assing Hvidt E, Sondergaard J, Ammentorp J, et al. The existential dimension in general practice: identifying understandings and experiences of general practitioners in Denmark. *Scandinavian Journal of Primary Health Care* 2016;34(4):385-93.

For peer review only

For peer review only - http://bmjopen.bmj.com/site/about/guidelines.xhtml

19. Holmes SM, Rabow MW, Dibble SL. Screening the soul: communication regarding spiritual concerns among primary care physicians and seriously ill patients approaching the end of life. *American Journal of Hospice & Palliative Medicine* 2006;23(1):25-33.
20. Saguil A, Phelps K. The spiritual assessment. *American Family Physician* 2012;86(6):546-50.
21. Monroe MH, Bynum D, Susi B, et al. Primary care physician preferences regarding spiritual behavior in medical practice. *Archives of Internal Medicine* 2003;163(22):2751-6.
22. McCauley J, Jenckes MW, Tarpley MJ, et al. Spiritual beliefs and barriers among managed care practitioners. *Journal of Religion & Health* 2005;44(2):137-46.
23. Larimore WL. Providing basic spiritual care for patients: Should it be the exclusive domain of pastoral professionals? *American Family Physician* 2001;63(1):36-40.
24. Daaleman TP, Nease DE, Jr. Patient attitudes regarding physician inquiry into spiritual and religious issues. *Journal of Family Practice* 1994;39(6):564-8.
25. Brennan M, Heiser D. Introduction: Spiritual Assessment and Intervention: Current Directions and Applications. *Journal of Religion, Spirituality & Aging* 2004;17(1-2):1-20. doi: 10.1300/J496v17n01_01
26. Lee-Poy M, Stewart M, Ryan BL, et al. Asking patients about their religious and spiritual beliefs: Cross-sectional study of family physicians. *Canadian Family Physician*;62(9):e555-61.
27. Appleby A, Swinton J, Bradbury I, et al. GPs and spiritual care: signed up or souled out? A quantitative analysis of GP trainers' understanding and application of the concept of spirituality. *Education for Primary Care* 2018;29(6):367-75.
28. Best M, Butow P, Olver I. Do patients want doctors to talk about spirituality? A systematic literature review. *Patient Education and Counseling* 2015;98(11):1320-28. doi: https://doi.org/10.1016/j.pec.2015.04.017
29. Puchalski CM, Vitillo R, Hull SK, et al. Improving the spiritual dimension of whole person care: reaching national and international consensus. *J Palliat Med* 2014;17(6):642-56. doi: 10.1089/jpm.2014.9427 [published Online First: 2014/05/19]
30. Guest G, MacQueen KM, Namey EE. Applied thematic analysis 'Themes and Codes' chapter. Thousand Oaks, CA: SAGE 2012.
31. Appleby A, Swinton J, Wilson P. What GPs mean by 'spirituality' and how they apply this concept with patients: a qualitative study. *BJGP open* 2018; 2(2). (accessed 2018/07//).
32. Borneman T, Ferrell B, Puchalski CM. Evaluation of the FICA Tool for Spiritual Assessment. *Journal of Pain and Symptom Management* 2010;40(2):163-73. doi: 10.1016/j.jpainsymman.2009.12.019
33. Lucchetti G, Bassi RM, Lucchetti ALG. Taking Spiritual History in Clinical Practice: A Systematic Review of Instruments. *EXPLORE: The Journal of Science and Healing* 2013;9(3):159-70. doi: https://doi.org/10.1016/j.explore.2013.02.004
34. Puchalski CM. Integrating spirituality into patient care: an essential element of person-centered care. *Polskie Archiwum Medycyny Wewnetrznej* 2013;123(9):491-97.
35. Koenig HG. Religion, Spirituality, and Health: The Research and Clinical Implications. *ISRN Psychiatry* 2012;2012:278730. doi: 10.5402/2012/278730
36. Assing Hvidt E, Søndergaard J, Ammentorp J, et al. The existential dimension in general practice: identifying understandings and experiences of general practitioners in Denmark. *Scandinavian Journal of Primary Health Care* 2016;34(4):385-93. doi: 10.1080/02813432.2016.1249064
37. The General Medical Council. What Our Data Tells us about General Practitioners working for the NHS in England and Scotland, 2018.
38. Brandling J, House W. Social prescribing in general practice: adding meaning to medicine. *British Journal of General Practice* 2009;59(563):454. doi: 10.3399/bjgp09X421085
39. Puchalski CM. The FICA Spiritual History Tool #274. *Journal of Palliative Medicine* 2014;17(1):105-6.

For peer review only - http://bmjopen.bmj.com/site/about/guidelines.xhtml

40. Paal P, Helo Y, Frick E. Spiritual Care Training Provided to Healthcare Professionals: A Systematic Review. *Journal of Pastoral Care & Counseling* 2015;69(1):19-30. doi: 10.1177/1542305015572955
41. Mueller PS, Plevak DJ, Rummans TA. Religious involvement, spirituality, and medicine: implications for clinical practice. *Mayo Clinic Proceedings* 2001;76(12):1225-35.
42. Larimore WL, Parker M, Crowther M. Should clinicians incorporate positive spirituality into their practices? What does the evidence say? *Annals of Behavioral Medicine* 2002;24(1):69-73.

For peer review only

For peer review only - http://bmjopen.bmj.com/site/about/guidelines.xhtml

Standards for Reporting Qualitative Research (SRQR)*

http://www.equator-network.org/reporting-guidelines/srqr/

Title and abstract

	Page/line no(s).
**Title** - Concise description of the nature and topic of the study Identifying the study as qualitative or indicating the approach (e.g., ethnography, grounded theory) or data collection methods (e.g., interview, focus group) is recommended	0
**Abstract** - Summary of key elements of the study using the abstract format of the intended publication; typically includes background, purpose, methods, results, and conclusions	0

Introduction

**Problem formulation** - Description and significance of the problem/phenomenon studied; review of relevant theory and empirical work; problem statement	2, 3
**Purpose or research question** - Purpose of the study and specific objectives or questions	3

Methods

**Qualitative approach and research paradigm** - Qualitative approach (e.g., ethnography, grounded theory, case study, phenomenology, narrative research) and guiding theory if appropriate; identifying the research paradigm (e.g., postpositivist, constructivist/ interpretivist) is also recommended; rationale**	3, 4
**Researcher characteristics and reflexivity** - Researchers' characteristics that may influence the research, including personal attributes, qualifications/experience, relationship with participants, assumptions, and/or presuppositions; potential or actual interaction between researchers' characteristics and the research questions, approach, methods, results, and/or transferability	4
**Context** - Setting/site and salient contextual factors; rationale**	2,3
**Sampling strategy** - How and why research participants, documents, or events were selected; criteria for deciding when no further sampling was necessary (e.g., sampling saturation); rationale**	1, 4
**Ethical issues pertaining to human subjects** - Documentation of approval by an appropriate ethics review board and participant consent, or explanation for lack thereof; other confidentiality and data security issues	10
**Data collection methods** - Types of data collected; details of data collection procedures including (as appropriate) start and stop dates of data collection and analysis, iterative process, triangulation of sources/methods, and modification of procedures in response to evolving study findings; rationale**	3, 4

For peer review only

For peer review only - http://bmjopen.bmj.com/site/about/guidelines.xhtml

**Data collection instruments and technologies** - Description of instruments (e.g., interview guides, questionnaires) and devices (e.g., audio recorders) used for data collection; if/how the instrument(s) changed over the course of the study	3
**Units of study** - Number and relevant characteristics of participants, documents, or events included in the study; level of participation (could be reported in results)	4 (table 1)
**Data processing** - Methods for processing data prior to and during analysis, including transcription, data entry, data management and security, verification of data integrity, data coding, and anonymization/de-identification of excerpts	3, 4
**Data analysis** - Process by which inferences, themes, etc., were identified and developed, including the researchers involved in data analysis; usually references a specific paradigm or approach; rationale**	3,4
**Techniques to enhance trustworthiness** - Techniques to enhance trustworthiness and credibility of data analysis (e.g., member checking, audit trail, triangulation); rationale**	4, 5, 6, 7 (table 2)

Results/findings

**Synthesis and interpretation** - Main findings (e.g., interpretations, inferences, and themes); might include development of a theory or model, or integration with prior research or theory	5, 6, 7 (table 2)
**Links to empirical data** - Evidence (e.g., quotes, field notes, text excerpts, photographs) to substantiate analytic findings	4, 5, 6, 7, (table 2)

Discussion

**Integration with prior work, implications, transferability, and contribution(s) to the field** - Short summary of main findings; explanation of how findings and conclusions connect to, support, elaborate on, or challenge conclusions of earlier scholarship; discussion of scope of application/generalizability; identification of unique contribution(s) to scholarship in a discipline or field	8
**Limitations** - Trustworthiness and limitations of findings	1

Other

**Conflicts of interest** - Potential sources of influence or perceived influence on study conduct and conclusions; how these were managed	10
**Funding** - Sources of funding and other support; role of funders in data collection, interpretation, and reporting	10

*The authors created the SRQR by searching the literature to identify guidelines, reporting standards, and critical appraisal criteria for qualitative research; reviewing the reference lists of retrieved sources; and contacting experts to gain feedback. The SRQR aims to improve the transparency of all aspects of qualitative research by providing clear standards for reporting qualitative research.

For peer review only - http://bmjopen.bmj.com/site/about/guidelines.xhtml

**The rationale should briefly discuss the justification for choosing that theory, approach, method, or technique rather than other options available, the assumptions and limitations implicit in those choices, and how those choices influence study conclusions and transferability. As appropriate, the rationale for several items might be discussed together.

Reference:

O'Brien BC, Harris IB, Beckman TJ, Reed DA, Cook DA. **Standards for reporting qualitative research: a synthesis of recommendations.** *Academic Medicine*, Vol. 89, No. 9 / Sept 2014 DOI: 10.1097/ACM.0000000000000388

For peer review only

For peer review only - http://bmjopen.bmj.com/site/about/guidelines.xhtml
